# Screening for colorectal cancer with immunological FOBT

**DOI:** 10.1038/sj.bjc.6601798

**Published:** 2004-04-13

**Authors:** S Ottó, L Döbrössy

**Affiliations:** 1 National Institute of Oncology, Orszagos Onkologiai Intezet, PO Box 21, Budapest H-1525, Hungary

**Sir**,

The article of Nakajima *et al* (2003) in the Journal addresses a problem of major public health importance, concluding that screening programme with immunochemical faecal occult blood test (FOBT) can be effective for prevention of advanced colorectal cancer. However, the screening method applied in their case–control study (i.e. the immunochemical haemagglutination method described by Saito *et al* (1995) may direct the attention to the rather controversial area of which is the optimal screening tool, if any, for early detection of colorectal cancer and its precursors.

Recently, the scientific and clinical evidences for the use of the available colorectal cancer-screening tests were critically assessed (Walsh and Terdiman, 2003a, 2003b). Supported by a large body of direct and indirect evidence, those review articles concluded recommending that all asymptomatic men and women over 50 years of age and older should undergo screening; however, it stated, ‘at present, the available evidence does not support choosing any test over the others’. In the same time, after having reviewed the available evidence, a special advisory group appointed by the American Cancer Society highlighted immunological stool testing as the *only exception* from its general conclusion that this time there is insufficient evidence to recommend the other emerging technologies for routine colorectal cancer screening (Levin *et al*, 2003).

Since the effectiveness of FOBT has been established by randomised controlled trials (Mandel *et al*, 1993; Mendel *et al*, 1999; Kronborg *et al*, 1996; Hardcastle *et al*, 1996), several tests based on peroxidase activity and guaiac-type reagents for colour reaction are most widely used on a public health scale, being noninvasive, simple and cheap methods, However, these reagents are not human-specific, because they may give positive reaction even with the peroxidase of several food stuffs, both of plant and animal origin (Cole and Young, 1999). These shortcomings result in an unacceptably high false positive rate.

To eliminate these shortcomings, a number of combined methods consisting of a guaiac test and an immunochemical test have been developed. In addition to the increased specificity, the immunochemical FOBTs have the advantage of not requiring dietary or drug restrictions, therefore are more patient-friendly, and more suitable for mass application. In recent years, several such immunological techniques have developed (Ottó and Eckhardt, 2000).

However, several problems may reduce the efficiency of these methods (St John *et al*, 1993; Ottó and Eckhardt, 2000). Most of these immunological techniques are usually limited to the demonstration of haemoglobin in the faeces. For example, depending on the site of bleeding, a certain degradation of haemoglobin may take place, or, faecal haemoglobin may become irreversibly bound to special components of the faeces. To eliminate these potential sources of error, some investigators have suggested the demonstration of additional proteins, more stable in the faeces than haemoglobin, as possible new indicators of intestinal bleeding.

We ourselves have developed a combined electrophoretic–immunoprecipitation technique of high sensitivity (2 *μ*g ml^−1^), suitable for simultaneous demonstration of two blood proteins, that is, haemoglobin and albumin, in the faecal sample (Ottó and Németh, 1993). The technique is simple, inexpensive and suitable for the reliable detection of occult blood. The parallel demonstration of the two proteins significantly enhanced the detectability of symptomless colorectal cancer (Ottó and Német, 1996). Furthermore, using immune sera raised against native haemoglobin and its fragments, as a differential diagnostic method, our technique is suitable for parallel demonstration of haemoglobin and its fragments; in this way, the origin of the bleeding can be localised (Ottó *et al*, 1995).

The modified two-phase (guaiac-type and immunochemical) faecal occult blood test (FECA-TEST) was used (without diet) as a screening tool in a pilot population-screening project for early detection of colorectal cancer. The project was carried out in 1997–1998 in a well-defined administrative area of the Capital, Budapest, Hungary (where – like in many other developed countries – colorectal cancer is the second leading cause of cancer death; therefore, screening for colorectal cancer is a major public health concern) with support from the World Bank ‘Close the gap’ public health programme. The purpose of the project was to demonstrate the feasibility and validity of the test is screening context, in addition to social acceptance and impact on early cure.

In total, 21 945 persons of the target population received the sample collecting containers, distributed by the family physicians, and 6805 persons had returned it, thus the compliance rate was 31%. A total of 292 samples were technically unsatisfactory. The mean age of those tested was 64 years (49–86); 6513 samples were analysed at the Central Clinical Laboratory, National Institute of Oncology (NIO), Budapest. The very sensitive colour reaction was positive in 1892 cases (29% of all tested); in the second phase, the immunochemical Hgb and Alb reactions were positive (5.8% of all tests performed). The test-positive cases were referred for colonoscopic assessment to the NIO. Colonoscopy was performed on 243 persons (65% of those referred; the rest was lost for the follow-up). Colorectal cancer was detected and histologically confirmed in 12 cases (5% of all test positives); six out of 12 cancer cases proved to be in early stage (Dukes A). In additional 59 cases (20%), adenomatous polyps were found. In the 2-year follow-up period, seven cancer cases were diagnosed among those test-positive cases who rejected the endoscopic assessment. The parallel demonstration of the two proteins significantly enhanced the detectability of colorectal cancer.

We are convinced that, at present, regular screening of population at average risk, using the combined immunological FOBT as screening tool, is the most promising way of reducing the colorectal cancer burden, one of the leading causes of cancer death in most of the developed countries worldwide.
